# Genetic Screening of Haploid Neural Stem Cells Reveals that *Nfkbia* and *Atp2b4* are Key Regulators of Oxidative Stress in Neural Precursors

**DOI:** 10.1002/advs.202309292

**Published:** 2024-04-26

**Authors:** Shaochen Nie, Wenhao Zhang, Xin Jin, Xiaoyan Li, Shengyi Sun, Yiding Zhao, Qingshen Jia, Luyuan Li, Yan Liu, Dayong Liu, Qian Gao

**Affiliations:** ^1^ Tianjin Key Laboratory of Oral Soft and Hard Tissues Restoration and Regeneration, Tianjin Medical University School of stomatology Tianjin Medical University School of Stomatology Tianjin 300070 China; ^2^ State Key Laboratory of Medicinal Chemical Biology and College of Pharmacy Nankai University Tianjin 300350 China; ^3^ School of Medicine Nankai University Tianjin 300071 China; ^4^ Department of Obstetrics Tianjin First Central Hospital Nankai University Tianjin 300192 China; ^5^ Tianjin Key Laboratory of Animal and Plant Resistance College of Life Sciences Tianjin Normal University Tianjin 300387 China

**Keywords:** *Atp2b4*, H_2_O_2_, HaNSCs, *Nfkbia*, oxidative stress

## Abstract

Neurological diseases are expected to become the leading cause of death in the next decade. Although little is known about it, the interaction between oxidative stress and inflammation is harmful to the nervous system. To find an advanced tool for neural genetics, mouse haploid neural stem cells (haNSCs) from the somite of chimeric mouse embryos at E8.5 is established. The haNSCs present a haploid neural progenitor identity for long‐term culture, promising to robustly differentiate into neural subtypes and being able to form cerebral organoids efficiently. Thereafter, haNSC mutants via a high‐throughput approach and screened targets of oxidative stress is generated using the specific mutant library. Deletion of *Nfkbia* (the top hit among the insertion mutants) reduces damage from reactive oxygen species (ROS) in NSCs exposed to H_2_O_2_. Transcriptome analysis revealed that *Atp2b4* is upregulated significantly in *Nfkbia*‐null NSCs and is probably responsible for the observed resistance. Additionally, overexpression of *Atp2b4* itself can increase the survival of NSCs in the presence of H_2_O_2_, suggesting that *Atp2b4* is closely involved in this resistance. Herein, a powerful haploid system is presented to study functional genetics in neural lineages, shedding light on the screening of critical genes and drugs for neurological diseases.

## Graphical abstract

This work illustrates an in vivo strategy to derive haploid neural stem cells (haNSCs) from chimeric somite. The haNSCs show neural progenitor properties with a single genome, promising in neural subtypes and organoids. The genetic screening of haNSCs for anti‐H_2_O_2_ approaches that *Nfkbia* and *Atp2b4* are two key regulators of anti‐oxidative stress in neural progenitors and cerebral organoids.

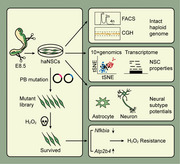

## 1 Introduction

Recently, neurodegenerative diseases have become one of the greatest threats to human health, and their incidence and influence have been widely studied. The exact cause of these diseases is complicated and difficult to identify; therefore, a convenient platform for neural development and pathological mechanism studies is needed. Neural stem cells (NSCs) derived from the central nervous tissues of embryos can self‐renew and give rise to three major neural lineages, neurons, oligodendrocytes, and astrocytes, and thus, NSCs can serve as an ideal cell model for neural development research in vitro.^[^ ^1^ ^]^ The establishment of NSCs as a potential resource of neurons and glia was a landmark in neuroscience research in the last four decades, satisfying the need when the nervous system lacks regenerative ability.^[^ ^2^ ^]^ Nevertheless, traditional NSCs are diploid, and it is difficult to perform genome‐wide functional genetics studies due to the backup of allelic genes.

Interestingly, mammalian haploid cell lines have been widely used in many approaches for high‐throughput genetic screening to reveal key target genes.^[^ ^3^, ^4^, ^5^ ^]^ This is especially true for recessive traits, which raises the question of whether we can establish haploid NSCs (haNSCs) from embryonic neural tissues and facilitate functional genetics in neural lineages. Although two groups attempted to derive haploid NSC‐like cells (haNSCLCs) from haploid embryonic stem cells (ESCs) by differentiation approximately ten years ago, both of them failed due to unexpected diploidization.^[^ ^3^, ^6^ ^]^ Due to the breakthroughs in introducing chemical compounds to maintain haploidy during culture,^[^ ^7^, ^8^, ^9^ ^]^ haploid differentiated heterogeneous NSCLCs and neurons can be obtained.^[^ ^8^, ^10^ ^]^ However, the effects of inhibitors are variable between batches, and these compounds rarely work well to prevent diploidization during differentiation in vivo.^[^ ^11^ ^]^ To the best of our knowledge, no haNSCs have been generated from neural tissues in vivo to date. Gene editing seemed to be a more efficient strategy to maintain haploidy in cell cultures,^[^ ^12^, ^13^ ^]^ and this method has been tested in chimera production experiments.^[^ ^14^, ^15^ ^]^ Whether these gene editing methods can promote the generation of haNSCs from in vivo neural tissues with improved homogeneity warrants more investigation. In addition, whether haNSCs are advanced in targeting critical genes in a desired screening is also fascinating. Oxidative stress is recognized as a pivotal regulatory factor in aging and various neurological disorders.^[^ ^16^ ^]^ As an organ that consumes a large amount of oxygen, the brain is particularly susceptible to the influence of oxidative stress, which potentially compromises the function of the central nervous system. The relationship between oxidative stress and neurodegenerative and neuropsychiatric disorders has raised extensive concerns, whereas the precise underlying mechanisms remain incompletely elucidated.^[^ ^17^ ^]^ Hence, it is critical to address the potential targets of oxidative stress in neural lineages on a global scale.

In this study, we attempt to derive haNSCs from somites of chimeric embryos at embryonic day 8.5 (E8.5) and characterize the identities of these specific haploid stem cells at different cellular and molecular levels. Thereafter, we assess the feasibility of using haNSCs for the genome‐wide screening of targets of oxidative toxicity and discuss potential key genes and the related mechanisms involved in oxidative stress in neural lineages.

## 2 Results

### 2.1 HaNSCs are derived from Chimeric Somite at E8.5

**Figure 1:**
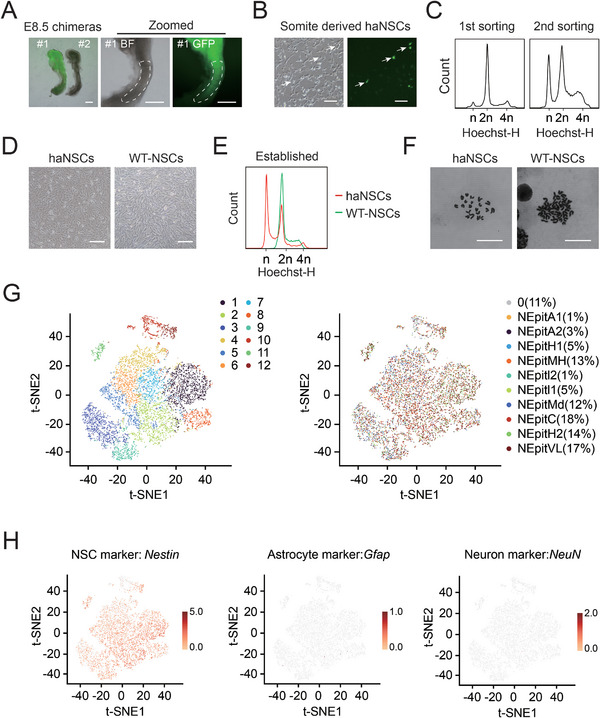
Derivation of haNSCs from somite. A) Images of chimeric embryos at E8.5 derived from *BCL2*‐OE haESCs as donor cells. GFP was visualized in the developing brain and somite. Scale bar, 100 µm. B) Phase‐contrast images of cell cultures from E8.5 chimeric somite tissue in the bright field and FITC channels showing typical neural progenitor cell morphology. Some cells were GFP‐positive, whereas the others were GFP‐negative. Scale bar, 100 µm. C) Analysis of DNA content in haNSCs at the first and second sorting. The percentages of the 1n peak (G0/G1) in haNSCs were 5.4% and 20.8%, respectively. D) Phase‐contrast images of haNSCs and WT‐NSCs. These cells were identical morphologically. Scale bar, 100 µm. E) DNA content profile of the established haNSCs. The percentage of the 1n peak (G0/G1) was 39.3%. A line of diploid WT‐NSCs was used as a control. F) Chromosome spreads of haNSCs and WT‐NSCs. haNSCs had a 20‐chromosome set, whereas WT‐NSCs showed 40 chromosomes in a single spread. Scale bar, 10 µm. G) t‐SNE plot showing the 12 main clusters of haNSCs. H) The expression levels of marker genes for NSCs and neural subtypes in haNSCs.

A previous report showed that mouse haESCs could contribute to chimeric embryos up to E10.5 stage by overexpressing *BCL2*.^[^ ^18^ ^]^ Thus, we tried to derive haNSCs from these chimeric embryos (Figure S1A, Supporting Information). We microinjected GFP‐labeled *BCL2*‐OE haESCs (Figure S1B, Supporting Information) into WT blastocysts and transferred these reconstructed embryos into the oviducts of pseudopregnant mice. Chimeric embryos at E8.5 showed the standard developmental morphology with the contribution from GFP and were dissected to retrieve somite tissues. The isolated spines that presented green fluorescence (**Figure** **1**A) were dissociated into single cells with trypsin, plated in Matrigel precoated plates and cultured in mNSC medium.^[^ ^19^ ^]^ Three to five days later, the somite cell cultures were morphologically similar to typical NSCs, some of which were GFP‐positive (Figure 1B). Although exogenic *BCL2*‐GFP OE was gradually silenced (Figure S1C, Supporting Information), we enriched the haploid cells according to the DNA content when the NSCs reached 90% confluence (Figure 1C). We found that haNSCs not only self‐renewed with a neural progenitor cell morphology in long‐term culture but also steadily maintained haploidy during periodic sorting (Figure 1D–F). Next, we performed genomic sequencing and RNA‐seq to analyze the properties of haNSCs. The results of CNV analysis showed that the haNSCs had an intact haploid genome with few mutations (Figure S1D, Supporting Information). In addition, the cluster dendrogram of RNA profiles showed that the haNSCs were similar to WT‐NSCs and haNSCLCs but vastly different from HaESCs and WT‐ESCs (Figure S1E, Supporting Information).

To further know the characteristics of haNSCs, we performed single‐cell RNA sequencing (scRNA‐seq) using the 10x Genomics technique. The results revealed a total of 9619 cells were extracted from our dataset, which were clustered into 12 populations based on the dendrogram. Notably, our data exhibited consistency with previously described single‐cell maps of mouse brain neural precursor cells (Figure 1G),^[^ ^20^ ^]^ manifesting their authentic NSC properties. The analysis of differential transcriptional states also revealed that most of haNSCs expressed NSC marker gene‐*Nestin*, whereas they expressed barely genes related to glial and neurons (Figure 1H). Taken together, these data show that these haNSCs could be derived from the somite of chimeras at E8.5 and were able to proliferate in a haploid NSC manner.

### 2.2 HaNSCs Present Neural Precursor Identities and Promise Differentiation into Neurons and Glia

**Figure 2:**
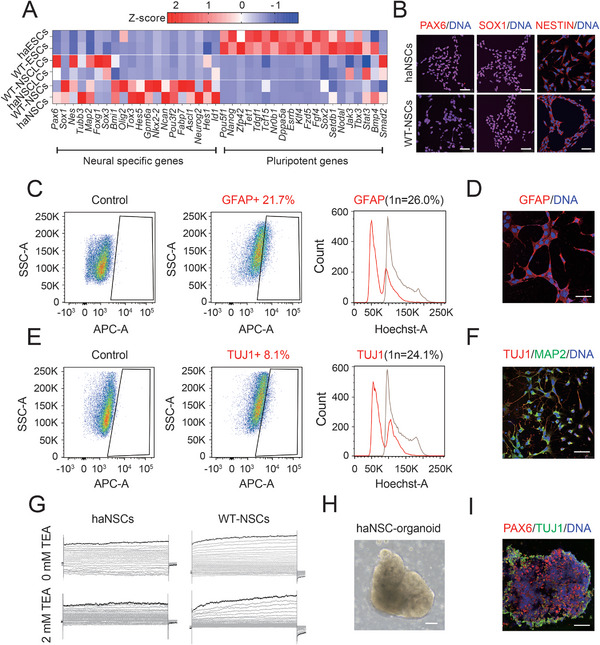
Identities of haNSCs and their neuronal differentiation. A) Heatmap of neural‐specific genes and pluripotent genes in haESCs, WT‐ESCs, WT‐NSCLCs, haNSCLCs, WT‐NSCs and haNSCs. B) Immunofluorescence staining of NSC‐specific markers (PAX6, SOX1 and NESTIN) in haNSCs and WT‐NSCs. DNA was stained with Hoechst 33342. Scale bar, 50 µm. C) FACS analysis of cell cultures differentiated from haNSCs for an astrocyte‐specific marker (GFAP) and DNA content. WT‐ESCs stained with the same flow cytometry antibody served as a control. D) Immunostaining of an astrocyte‐specific marker (GFAP) in differentiated cells from haNSCs. Scale bar, 100 µm. E) FACS analysis of cell cultures differentiated from haNSCs for a neuron‐specific marker (TUJ1) and DNA content. WT‐ESCs stained with the same flow cytometry antibody served as a control. F) Immunostaining of neuron markers (TUJ1 and MAP2) in differentiated cells from haNSCs. Scale bar, 100 µm. G) Electrophysiological assessment of haNSCs and WT‐NSCs. TEA was added to both haNSCs and WT‐NSCs to inhibit IV currents. H) Cerebral organoids derived from haNSCs in bright field. Scale bar, 100 µm. I) Immunofluorescence staining of NSC‐specific markers (PAX6 and TUJ1) in haNSC‐derived cerebral organoids. Scale bar, 50 µm.

To determine whether haNSCs possessed neural properties, we performed the following experiments. The heatmap results showed that the haNSCs highly expressed neural‐specific genes instead of pluripotent genes compared with WT‐NSCs, haESCs and other cell lines (**Figure** **2**A). In addition, the immunofluorescence results confirmed that the positive expression of the neural progenitor‐specific markers PAX6, SOX1 and NESTIN in the haNSCs using WT‐NSCs as a positive control (Figure 2B). Another characteristic of NSCs is that they can proliferate in floating aggregated neural spheres. Our haNSCs had the ability to robustly aggregate to form neural spheres (Figure S2A, Supporting Information) that expressed the neural progenitor‐specific marker genes *Pax6* and *Nestin*, as shown by qPCR, compared to WT‐ESCs and other neural spheres (Figure S2B, Supporting Information). To examine the neuronal differentiation potentials of haNSCs, we performed random differentiation to form astrocytes and Tuj1‐positive neurons. Interestingly, 26.0% of the cells remained haploid in the 1n peak of the GFAP‐positive (GFAP+, 21.7%) population (Figure 2C); the GFAP‐positive cell cultures were confirmed by immunostaining (Figure 2D). In another parallel experiment, there were 24.1% haploid cells in the 1n peak among the Tuj1‐positive (Tuj1+, 8.1%) population (Figure 2E), indicating that haploidy was durable in neurons. The cell cultures were confirmed to be positive for several neuronal markers, including TUJ1, MAP2, NEUN and SERT (Figures 2F; Figure S2C, Supporting Information).

To determine whether haNSCs had electrophysiological functions, we utilized the whole‐cell patch clamp technique to detect voltage‐gated outward currents. Briefly, both haNSCs and WT‐NSCs were exposed to depolarizing pulses ranging from −80 to +70 mV in increments of 10 mV. The experiments began with a holding potential of −60 mV. The results from the whole‐cell patch clamp tests revealed pronounced IV currents in both WT‐NSCs and haNSCs. The addition of the potassium channel blocker tetraethylammonium (TEA) (2 mm) markedly inhibited the IV currents in both haNSCs and WT‐NSCs (Figures 2G; Figure S2D, Supporting Information), which is a standard characteristic of functional neural cells. Self‐organizing to form 3D cerebral organoids was a typical identity of neural progenitor cells,^[^ ^21^ ^]^ but whether haNSCs could form such organoids was uncertain. Interestingly, our haNSCs could form 3D organoids expressing representative markers of PAX6 and TUJ1 (Figure 2H,I). In total, our haNSCs not only presented authentic NSC properties but also possessed differentiation potentials to neural subtypes in a haploid manner.

### 2.3 Genetic Screening of H_2_O_2_ Targets using haNSCs Identifies Multiple Candidate Genes

**Figure 3:**
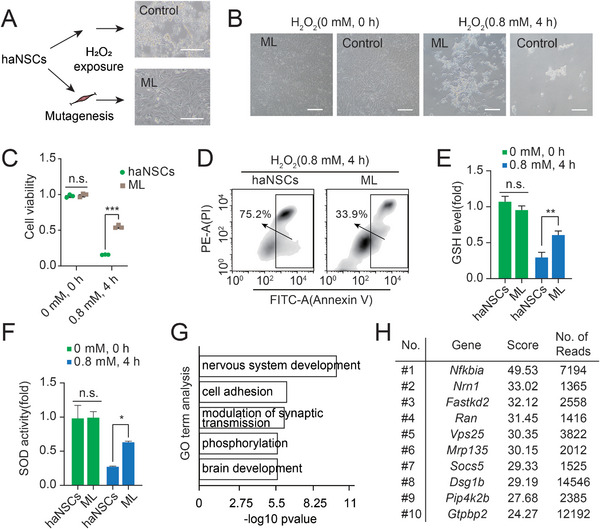
Genetic screening of haNSCs for oxidative toxicant resistant genes. A) Schematic overview of the screening for H_2_O_2_ resistance genes using mutant haNSCs (ML) with nonmutant WT‐haNSCs as a negative control. B) Phase‐contrast images of ML and control haNSCs after treatment with/without 0.8 mm H_2_O_2_ for 4 h. Scale bar, 100 µm. C) Assessment of the viability of ML and control haNSCs with/without 0.8 mm H_2_O_2_ treatment for 4 h according to CCK‐8 assay analysis. The data represented three replicates. t test, ***p < 0.001. Data were presented as the mean ± SD. D) Apoptosis analysis of ML and control haNSCs after treatment with 0.8 mm H_2_O_2_ for 4 h by PI/Annexin V assay. E) GSH levels in ML and control haNSCs with/without 0.8 mm H_2_O_2_ treatment for 4 h. Data represented three replicates. t test, **p < 0.01, n.s. not significant. Data were presented as the mean ± SD. F) SOD activities in ML and control haNSCs with/without 0.8 mm H_2_O_2_ treatment for 4 h. Data represented three replicates. t test, *p < 0.05, n.s. not significant. Data were presented as the mean ± SD. G) GO analysis of the top 1000 inserted genes analyzed from ML. H) The top 10 inserted genes among all mutations.

Given that haNSCs can expand in haploidy with neuronal potential, it was necessary to determine whether they had advantages in the discovery of oxidative toxicant target genes of neural lineages. First, we employed a designed *PiggyBac* (PB)‐trapping vector (Figure S3A, Supporting Information) to induce whole‐genome mutations in haNSCs. Hydrogen peroxide (H_2_O_2_) is a widely used oxidative stress inducer that is very harmful to cells, and it was chosen to screen a resistant mutant library (ML) (**Figure** **3**A). An optimized lethal concentration of H_2_O_2_ and time of treatment (0.8 mm, 4 h) were determined in a preliminary experiment (Figure S3B, Supporting Information). Next, we treated mutant cells and WT‐haNSCs (nonmutant control) with 0.8 mm H_2_O_2_ for 4 h. The results revealed that all of the WT‐haNSCs died after H_2_O_2_ treatment, whereas some cells in the ML group survived and could expand after H_2_O_2_ withdrawal (Figure 3B). Furthermore, both the CCK8 assay and PI/Annexin V assay results confirmed that the ML group presented a better survival ability after exposure to H_2_O_2_ (Figure 3C,D). Subsequently, we assessed the levels of GSH and the activities of SOD in ML and WT‐haNSCs and found that the ML group showed increased GSH levels (Figure 3E) and SOD activities (Figure 3F) in the presence of H_2_O_2_ compared with those in WT‐haNSCs. All these results demonstrated that the ML NSCs that had survived our screening system possessed notable resistance to oxidative damage.

Thereafter, we performed next‐generation sequencing (NGS) to analyze the insertions of the ML cells. Among the integrated genes in the ML cells, 58.89% of the insertions occurred in the intron regions, 0.76% in the 3′‐UTR regions, 0.02% in the 5′‐UTR regions, 2.28% in the exon regions, and 5.34% in the promoter regions (Figure S3C, Supporting Information). Gene Ontology (GO) analysis indicated that the top 1000 inserted genes were associated with the regulation of nervous system development, cell adhesion, and other functions (Figure 3G). The top 10 inserted genes were ranked according to frequency and software score and analyzed in further investigations (Figure 3H).

### 2.4 Deletion of Nfkbia Enhances the Antioxidant Ability of NSCs

**Figure 4:**
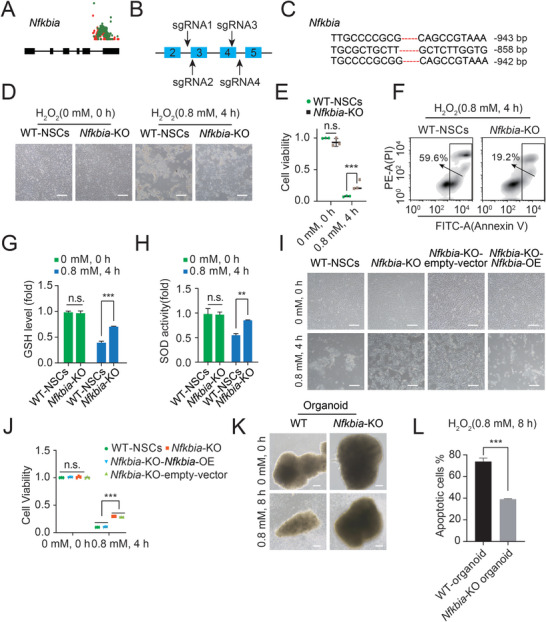
Validation of the involvement of Nfkbia‐KO in oxidative toxicity resistance. A) Insertions of *Nfkbia* are depicted with sense (red) and antisense (blue) orientations. The rectangles represent the exons of *Nfkbia*. B) Schematic of the design of *Nfkbia*‐KO based on the CRISPR/Cas9 system. C) Genotype sequencing results of *Nfkbia*‐KO NSCs. D) Phase‐contrast images of WT‐NSCs and *Nfkbia*‐KO NSCs treated with/without 0.8 mm H_2_O_2_ for 4 h. Scale bar, 100 µm. E) CCK‐8 assay results with WT‐NSCs and *Nfkbia*‐KO NSCs after treatment with 0.8 mm H_2_O_2_ for 4 h. Data represented three replicates. t test, ***p < 0.001. Data were presented as the mean ± SD. F) Apoptosis analysis of WT‐NSCs and *Nfkbia*‐KO NSCs after treatment with 0.8 mm H_2_O_2_ for 4 h by PI/Annexin V assay. G) GSH levels in WT‐NSCs and *Nfkbia*‐KO NSCs after treatment with/without 0.8 mm H_2_O_2_ for 4 h. Data represented three replicates. t test, ***p < 0.001, n.s. not significant. Data were presented as the mean ± SD. H) SOD activities in WT‐NSCs and *Nfkbia*‐KO NSCs after treatment with/without 0.8 mm H_2_O_2_ for 4 h. Data represented three replicates. t test, **p < 0.01, n.s. not significant. Data were presented as the mean ± SD. I) Phase‐contrast images of WT‐NSCs, *Nfkbia*‐KO NSCs, *Nfkbia‐*KO‐empty vector NSCs and *Nfkbia*‐KO‐*Nfkbia*‐OE NSCs after treatment with/without 0.8 mm H_2_O_2_ for 4 h. Scale bar, 100 µm. J) CCK‐8 assay results with WT‐NSCs, *Nfkbia*‐KO NSCs*, Nfkbia‐*KO‐empty vector NSCs and *Nfkbia*‐KO‐*Nfkbia*‐OE NSCs after treatment with 0.8 mm H_2_O_2_ for 4 h. The data represented three replicates. t test, ***p < 0.001. Data were presented as the mean ± SD. K) Phase‐contrast images of WT‐organoids and *Nfkbia*‐KO organoids treated with/without 0.8 mm H_2_O_2_ for 8 h. Scale bar, 100 µm. L) Apoptosis analysis of WT‐organoids and *Nfkbia*‐KO organoids after treatment with 0.8 mm H_2_O_2_ for 8 h by DRAQ7 analysis. Data represented three replicates. t test, ***p < 0.001, n.s. not significant. Data were presented as the mean ± SD.

Now that the inserted genes involved in the anti‐oxidative toxicity of NSCs were uncovered, it was important to perform proof‐of‐concept validations. To validate the function of the screened inserted genes, we chose the top gene, *Nfkbia*, to perform gene knockout (KO) and subsequent experiments. *Nfkbia* (Nuclear Factor of Kappa Light Polypeptide Gene Enhancer in B Cells Inhibitor, Alpha) carried the most PB insertions, as indicated in **Figure** **4**A. We designed two pairs of sgRNAs to target *Nfkbia* and constructed specific CRISPR/Cas9 plasmids (Figure 4B). Next, we electroporated *Nfkbia*‐KO plasmids into WT‐NSCs and enriched RFP‐positive cells by FACS to ensure transfection. Both the genotyping and Western blotting (WB) results indicated the disruption of *Nfkbia* in WT‐NSCs (Figure S4A–C, Supporting Information). Then, the same number of *Nfkbia*‐KO NSCs and WT‐NSCs were exposed to 0.8 mm H_2_O_2_ for 4 h to detect their antioxidant abilities. After H_2_O_2_ withdrawal, we observed that some cells in the *Nfkbia*‐KO NSC group survived and could be expanded for further culture, whereas all of the cells in the WT‐NSC group died after H_2_O_2_ treatment (Figure 4D). Both the CCK8 assay and Annexin V/PI assay results demonstrated that *Nfkbia*‐KO NSCs presented better viability than WT‐NSCs after H_2_O_2_ treatment (Figure 4E,F). Given that GSH and SOD are the most crucial antioxidants in cell cultures and that their levels represent the antioxidant ability of the cells, we assessed the GSH levels and SOD activities in *Nfkbia*‐KO NSCs and WT‐NSCs after exposure to H_2_O_2_. The results revealed that *Nfkbia*‐KO NSCs showed increased GSH levels and SOD activities compared with WT‐NSCs after being treated with H_2_O_2_ (Figure 4G,H). To further confirm this conclusion, we performed a rescue experiment by introducing overexpression (OE) of *Nfkbia* in *Nfkbia*‐KO NSCs (termed *Nfkbia*‐KO‐*Nfkbia*‐OE NSCs) (Figure S4C, Supporting Information). Notably, *Nfkbia*‐KO‐*Nfkbia*‐OE NSCs no longer showed antioxidant ability after exposure to H_2_O_2_ (Figure 4I), indicating that the re‐expression of *Nfkbia* made NSCs vulnerable to H_2_O_2_. Next, we examined the viability of and GSH level and SOD activity in *Nfkbia*‐KO‐*Nfkbia*‐OE NSCs and found that these cells showed reduced viability, GSH levels and SOD activities compared with *Nfkbia*‐KO NSCs after treatment with H_2_O_2_ (Figures 4J; Figure S4D–F, Supporting Information). Furtherly, we checked the anti‐oxidative stress ability of *Nfkbia*‐KO organoids and WT organoids (Figure 4K). The DRAQ7 analysis showed that the *Nfkbia*‐KO organoids had less apoptotic cells than those in WT organoids (Figure 4L). These data further confirmed that *Nfkbia* played a critical role in oxidative stress in NSCs. Overall, all the evidence suggested that disruption of *Nfkbia* could greatly enhance the antioxidative activity of NSCs.

### 2.5 Nfkbia‐Null Activates Ion Signaling‐Related Genes, Including Atp2b4, to Resist H_2_O_2_

**Figure 5:**
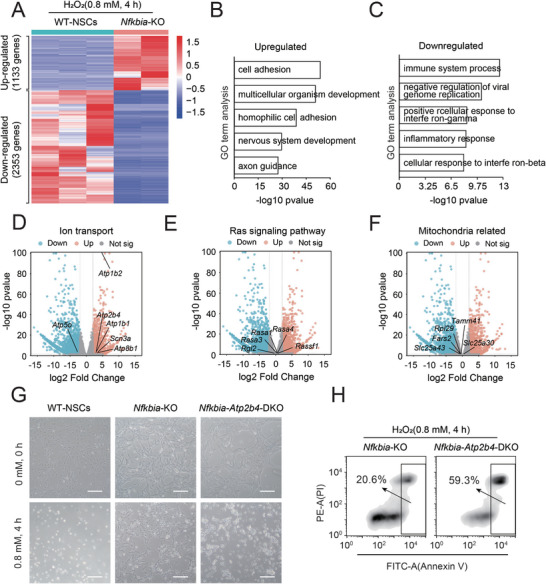
Analysis of Nfkbia‐related genes involved in oxidative stress. A) Heatmap of DEGs among *Nfkbia*‐KO NSCs and WT‐NSCs after treatment with 0.8 mm H_2_O_2_ for 4 h. B) GO analysis of the upregulated genes in *Nfkbia*‐KO NSCs compared with WT‐NSCs after exposure to 0.8 mm H_2_O_2_ for 4 h. C) GO analysis of the downregulated genes in *Nfkbia*‐KO NSCs compared with WT‐NSCs after exposure to 0.8 mm H_2_O_2_ for 4 h. D) Volcano plots showing the DEGs between *Nfkbia*‐KO NSCs and WT‐NSCs after treatment with 0.8 mm H_2_O_2_ for 4 h. Highlighted points indicate representative genes related to ion transport. E) Volcano plots showing the DEGs between *Nfkbia*‐KO NSCs and WT‐NSCs after treatment with 0.8 mm H_2_O_2_ for 4 h. Highlighted points indicate representative genes related to the ras signaling pathway. F) Volcano plots showing the DEGs between *Nfkbia*‐KO NSCs and WT‐NSCs after treatment with 0.8 mm H_2_O_2_ for 4 h. Highlighted points indicate representative genes related to mitochondria. G) Phase‐contrast images of WT‐NSCs, *Nfkbia*‐KO NSCs and *Nfkbia*‐*Atp2b4*‐DKO NSCs after treatment with/without 0.8 mm H_2_O_2_ for 4 h. Scale bar, 100 µm. H) Apoptosis analysis of *Nfkbia*‐KO NSCs and *Nfkbia*‐*Atp2b4*‐DKO NSCs after treatment with 0.8 mm H_2_O_2_ for 4 h by PI/Annexin V assay.

Given that *Nfkbia* was a key regulator for the anti‐oxidative toxicity of NSCs, it was necessary to investigate its underlying mechanism. To address this, we analyzed the global transcriptional profiles of *Nfkbia*‐KO NSCs and WT‐NSCs after treatment with H_2_O_2_ (0.8 mm, 4 h). According to the RNA sequencing (RNA‐seq) results, there were 1133 upregulated genes and 2353 downregulated genes in *Nfkbia*‐KO NSCs compared to WT‐NSCs (**Figure** **5**A). The upregulated genes in *Nfkbia*‐KO NSCs were mainly enriched in signaling pathways including cell adhesion and multicellular organism development, among others (Figure 5B). Moreover, the downregulated genes in *Nfkbia*‐KO NSCs were associated with pathways including immune system processes and negative regulation of viral genome replication, among others (Figure 5C). Furthermore, we analyzed the differentially expressed genes (DEGs) between *Nfkbia*‐KO NSCs and WT‐NSCs and mapped the DEGs to some representative pathways. Interestingly, some ion channel signaling‐related genes, including *Atp2b4*, showed remarkable upregulation in *Nfkbia*‐KO NSCs (Figure 5D). However, most Ras signaling‐related genes and mitochondria function‐related genes were located among the non‐significantly different genes between *Nfkbia*‐KO NSCs and WT‐NSCs (Figure 5E,F). *Atp2b4* (also known as PMCA4) encodes a calcium ion transport ATPase that plays an essential role in the regulation and transport of calcium ions both intra‐ and extracellularly.^[^ ^22^ ^]^ Thus, we hypothesized that *Atp2b4* might be a potential downstream regulator of *Nfkbia* in oxidative stress. Thereafter, we performed *Atp2b4* KO in *Nfkbia*‐KO NSCs (termed *Nfkbia‐Atp2b4*‐double KO (DKO) NSCs) by the CRISPR/Cas9 system (Figure S5A,B, Supporting Information) and found that *Nfkbia‐Atp2b4*‐DKO NSCs lost their antioxidant ability compared with *Nfkbia*‐KO NSCs under the same H_2_O_2_ treatment conditions (Figure 5G,H; Figure S5C,D, Supporting Information). In addition, *Nfkbia‐Atp2b4*‐DKO NSCs showed reduced GSH levels and SOD activities compared with *Nfkbia*‐KO NSCs after exposure to H_2_O_2_ (Figure S5E,F, Supporting Information). In order to further investigate the mechanism why *Nfkbia‐Atp2b4*‐DKO negatively regulated the anti‐oxidant injury in NSCs, we performed RNA‐Seq analysis. Following H_2_O_2_ treatment, it was observed that the *Nfkbia‐Atp2b4*‐DKO led to a significant upregulation of 3019 genes and downregulation of 2924 genes when compared to the *Nfkbia*‐KO group (Figure S5G, Supporting Information). Among these DEGs, the upregulated genes were mainly enriched in pathways related to nervous system development, neurogenesis, and others (Figure S5H, Supporting Information), while the downregulated genes were primarily associated with morphological changes in anatomical structure morphogenesis and other process (Figure S5I, Supporting Information). These DEGs and related pathways might be potential reason for role of *Atp2b4* in the Anti‐oxidative stress of *Nfkbia*‐KO. Altogether, above findings validated that *Atp2b4* played an important role in the antioxidant abilities of *Nfkbia*‐KO NSCs.

### 2.6 The Overexpression of Atp2b4 Itself Helps with Resistance to H_2_O_2_ in NSCs

**Figure 6:**
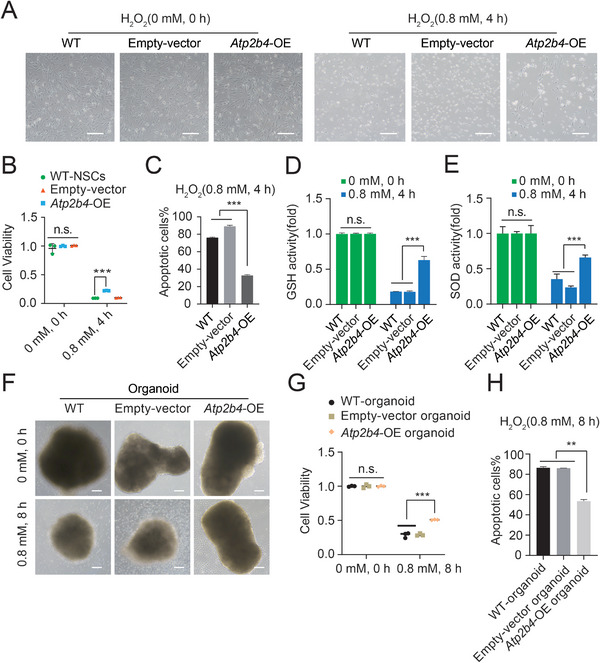
Overexpression of Atp2b4 in NSCs reduces H2O2‐induced oxidative stress. A) Phase‐contrast images of WT‐NSCs, empty‐vector NSCs and *Atp2b4*‐OE NSCs after treatment with/without 0.8 mm H_2_O_2_ for 4 h. Scale bar, 100 µm. B) CCK‐8 assay results with WT‐NSCs, empty‐vector NSCs and *Atp2b4*‐OE NSCs after treatment with/without 0.8 mm H_2_O_2_ for 4 h. The data represented three replicates. t test, ***p < 0.001. Data were presented as the mean ± SD. C) Apoptosis analysis of WT‐NSCs, empty‐vector NSCs and *Atp2b4*‐OE NSCs after treatment with 0.8 mm H_2_O_2_ for 4 h by DRAQ7 analysis. D) GSH levels in WT‐NSCs, empty‐vector NSCs and *Atp2b4*‐OE NSCs with/without 0.8 mm H_2_O_2_ treatment for 4 h. Data represented three replicates. t test, ***p < 0.001, n.s. not significant. Data were presented as the mean ± SD. E) SOD activities in WT‐NSCs, empty‐vector NSCs and *Atp2b4*‐OE NSCs after treatment with/without 0.8 mm H_2_O_2_ for 4 h. Data represented three replicates. t test, ***p < 0.001, n.s. not significant. Data were presented as the mean ± SD. F) Phase‐contrast images of WT‐organoid, empty‐vector organoid and *Atp2b4*‐OE organoid treated with/without 0.8 mm H2O2 for 8 h. Scale bar, 100 µm. G) CCK‐8 assay results with WT‐organoid, empty‐vector organoid and *Atp2b4*‐OE organoid after treatment with/without 0.8 mm H_2_O_2_ for 8 h. The data represented three replicates. t test, ***p < 0.001. Data were presented as the mean ± SD. H) Apoptosis analysis of WT‐organoid, empty‐vector organoid and *Atp2b4*‐OE organoid after treatment with 0.8 mm H_2_O_2_ for 8 h by DRAQ7 analysis. Data represented three replicates. t test, **p < 0.01, n.s. not significant. Data were presented as the mean ± SD.

*Atp2b4* is rarely studied in the oxidative damage response; however, it is interesting to determine whether *Atp2b4* itself can act as a modulator of oxidative stress resistance. To investigate the function of *Atp2b4*, we performed *Atp2b4*‐OE in WT‐NSCs by PB‐based vector delivery, using the loading vector as an empty control (Figure S6A, Supporting Information). The real‐time qPCR results revealed that the expression level of *Atp2b4* in the *Atp2b4*‐OE group was significantly higher than that in the WT‐NSC and empty‐vector NSC groups (Figure S6B, Supporting Information). After treatment with H_2_O_2_ (0.8 mm, 4 h), several NSCs in the *Atp2b4*‐OE group survived, whereas all of the WT‐NSCs and the cells in the empty vector group died (**Figure** **6**A). The CCK8 assay and DRAQ7 analysis results indicated that *Atp2b4*‐OE NSCs showed increased viability compared with WT‐NSCs and empty vector NSCs after exposure to H_2_O_2_ (Figure 6B,C). In addition, the increase in GSH levels and SOD activities in *Atp2b4*‐OE NSCs after treatment with H_2_O_2_ confirmed the role of *Atp2b4*‐OE following exposure to H_2_O_2_ (Figure 6D,E). We also checked the anti‐oxidative stress abilities of WT organoids, empty vector organoids and *Atp2b4*‐OE organoids (Figure 6F). The CCK8 assay and DRAQ7 analysis results indicated that *Atp2b4*‐OE organoids had more cells survived than those in WT organoids and empty vector organoids when exposed to H_2_O_2_ (Figure 6G,H). Overall, *Atp2b4*‐OE itself enhanced the resistance of NSCs to the oxidative toxicant H_2_O_2_. To further elucidate the underlying mechanism of H_2_O_2_ resistance in *Atp2b4*‐OE NSCs, we performed RNA‐seq analysis to compare the global transcriptional profiles of *Atp2b4*‐OE NSCs, *Nfkbia*‐KO NSCs and WT‐NSCs. Compared with *Nfkbia*‐KO NSCs, *Atp2b4*‐OE NSCs exhibited 2821 upregulated genes and 1797 downregulated genes, which were different from the DEGs in *Nfkbia*‐KO NSCs (Figure S6C,D, Supporting Information). According to the GO analysis, the upregulated genes in *Atp2b4*‐OE NSCs were mainly enriched in the functions of postsynaptic membrane, synaptic membrane and others (Figure S6E, Supporting Information), while the downregulated genes were mainly enriched in the functions of negative regulation of cellular process and others (Figure S6F, Supporting Information). These results suggested that the resistance of *Atp2b4*‐OE NSCs to oxidative stress might be associated with regulation of the postsynaptic membrane, synaptic membrane and others.

## 3 Discussion

Oxidative stress is intimately correlated with the onset of various maladies, particularly neurodegenerative disorders, which have been unequivocally intertwined with oxidative damage. Oxidative stress begets a cascade of events where free radicals associate with neural cells, culminating in erroneous protein folding, the activation of glial cells, compromised mitochondrial function, impaired DNA repair systems, and even cell death.^[^ ^23^ ^]^ However, there is still a lack of robust cellular tools to investigate the key genes involved in oxidative damage to neural lineages. Haploid cells, which feature a single set of chromosomes, in contrast to diploid WT‐NSCs, exhibit a heightened propensity for generating whole‐genome hemizygous mutations, thus holding an overwhelming advantage in genetic analysis and pharmaceutical screenings.^[^ ^11^ ^]^ Although haNSCLCs have already been derived from haESCs by differentiation in vitro,^[^ ^8^, ^24^ ^]^ these derivatives exhibited conspicuous heterogeneity, with variable and unpredictable differentiation potentials, which compromise their application in neural‐specific functional genetics. Therefore, deriving haNSCs from in vivo neural tissue with higher homogeneity and durable differentiation potentials to neurons and glia is both a great challenge and an urgent need. Our group previously found that *BCL2*‐OE strongly facilitated the maintenance of haploid populations, even in the efficient contribution of haESCs into E8.5 chimeric mice.^[^ ^18^ ^]^ Based on the same strategy, we derived a haNSC line from E8.5 chimeric somite tissue (Figure S1A, Supporting Information; Figure 1A). Our haNSCs had a stable haploid proportion and intact genome (Figure 1E–G). Furthermore, they exhibited characteristics and functionalities similar to WT‐NSCs (Figure 2). In addition, according to the RNA‐seq results, there were 1567 upregulated genes and 1509 downregulated genes in haNSCs when compared to haNSCLCs (Figure S7A, Supporting Information). The upregulated genes in haNSCs were mainly enriched in anatomical structure morphogenesis and others (Figure S7B, Supporting Information). While the downregulated genes in haNSCs were associated with system development, multicellular organism development and others (Figure S7C, Supporting Information). All the expression differences indicated that our haNSCs were different from previous reported haNSCLCs. Nevertheless, these features of haNSCs promised them advanced in the screening of target genes of neural lineages for any desired approach. However, whether this protocol is suitable for deriving haNSCs in other species still needs more investigation.^[^ ^25^ ^]^

As we have always performed genome‐wide mutations in haNSCs, we generated haNSC mutants via a high‐throughput approach by a PB‐based trapping strategy (Figure S3A, Supporting Information) and obtained H_2_O_2_‐resistant mutant NSCs (Figure 3A). From the ML NSCs, we identified multiple genes associated with oxidative damage in neural lineages, which were useful for research on oxidative stress resistance (Figure 3G,H). Next, we identified *Nfkbia* as a key regulator of oxidative stress in NSCs (Figure 4), which was reported to have the capacity to dimerize and bind to p65 and p50 and serve as a negative feedback regulator in the modulation of the NF‐κB signaling pathway.^[^ ^26^ ^]^ NF‐κB is a complex signaling pathway involved in regulating various biological responses. The precise role of *Nfkbia* in the oxidative stress response in neural lineages remains to be further clarified. This study primarily investigated the role of *Nfkbia* in the resistance of NSCs to H_2_O_2_ and the respective biological changes. We observed that *Nfkbia* KO led to an obvious increase in the expression of *Atp2b4* (Figure 5D), which encodes one of the four isoforms of p‐type ATPase (PMCA) and plays a key role in maintaining intracellular calcium homeostasis by actively extruding Ca^2+^ outside the cytoplasm.^[^ ^27^, ^28^ ^]^ Additionally, we found that *Nfkbia‐Atp2b4* DKO cells lose their resistance to oxidative damage (Figure 5G), demonstrating the necessary role of *Atp2b4* in the oxidative damage response of *Nfkbia*‐KO NSCs. Previous studies have explored the interaction between PMCA4b and neuronal nitric oxide synthase, which inhibits the production of nitric oxide (NO).^[^ ^29^ ^]^ Our results confirmed that *Atp2b4*‐OE in WT‐NSCs also enhances the ability of NSCs to resist oxidative damage (Figure 6A) due to the increased GSH levels and SOD activities compared with WT‐NSCs (Figure 6D,E).

In summary, we derived a haNSC cell line from somites in vivo that can steadily maintain haploid proportions and presents the characteristics and potential of NSCs. We conducted a H_2_O_2_ toxicity screening using haNSCs and identified several candidate genes associated with the oxidative stress response. We found that two critical regulators, *Nfkbia* and *Atp2b4*, play essential roles in the antioxidant abilities of NSCs, which represents a new approach to investigate oxidative stress mechanisms in the nervous system. Various neurological disorders are closely associated with oxidative damage.^[^ ^30^, ^31^, ^32^ ^]^ The application of haNSCs in whole‐genome genetic screening provides a robust platform for investigating neurodegenerative diseases and the developing targeted drugs for neurotoxic substances. Our findings offer new insights into the mechanisms underlying the onset of neurological disorders and hold promise for potential clinical applications as therapeutic targets.

## 4 Experimental Section and Subject Details

### Mice and Chimeric Embryo Reconstruction

The purchased specific pathogen‐free (SPF)‐grade mice (from Beijing Vital River Laboratory Animal Technology Co., Ltd. (Beijing, China)) were housed at the Nankai University Animal Center. All animal‐related experiments were performed according to the guidelines of the Animal Care and Use Committee of Nankai University.

### Cell Culture

*GFP‐*labeled *BCL2‐*OE parthenogenetic haESCs and other cell lines were previously established by the group.^[^ ^18^ ^]^ All ESCs were cultured in T2i medium consisting of DMEM/F12 (Thermo, 12500062, USA) supplemented with 15% fetal bovine serum (HyClone, SH30406.05, USA), 1% nonessential amino acids (Thermo, 11140050, USA), 0.1 mm β‐mercaptoethanol, 10 µg mL^−1^ penicillin‒streptomycin (Thermo, 15140122, USA), 1000 U mL^−1^ leukemia inhibitory factor (Millipore, ESG1107, USA), 0.2 µm PD0325901 (MCE, HY10254, China) and 3 µm CHIR99021 (MCE, HY10182, China). ESCs were passaged with 0.25% trypsin‐EDTA (Thermo, 25200072, USA) every other day.All NSCs and NSCLCs were expanded in NSC medium (Ndiff medium (Takara, Y40002, Japan) supplemented with 10 ng mL^−1^ mouse EGF (PeproTech, 31509, USA) and 10 ng mL^−1^ bFGF (PeproTech, 100–18B, USA) and differentiated in Ndiff medium only.

### Derivation of Mouse haNSCs

Somites dissected from E8.5 chimeric embryos (*GFP‐*labeled *BCL2*‐OE haESCs were used as the donor cells) were used for haNSC derivation. Briefly, *GFP‐*labeled *BCL2*‐OE haESCs were trypsinized into single cells and incubated with 3 µg mL^−1^ Hoechst 33342 (Thermo. H3570, USA) at 37 °C for 25 min and sorted with a cell sorter (Beckman, MoFloAstrios EQ, USA) to select for *GFP‐*labeled haploid cells. These GFP‐haploid cells were microinjected into CD‐1 background blastocysts to reconstruct chimeric embryos, which were transferred to the oviducts of pseudopregnant mice at 0.5 d.p.c.

### Isolation of haNSCs and WT‐NSCs

E8.5 embryos (chimera or WT) were dissected and dissociated with 0.05% trypsin‐EDTA (Thermo, 25200062, USA). The prepared cells were plated onto Matrigel (BD, 354230, USA) precoated plates (one well of a 24‐well plate each (NEST, 702001, China)) and cultured in NSC medium. During NSC culture, half of the medium changed every day, and the cells were passaged every 3–5 days. For haNSC enrichment, haploid cells were periodically sorted every 1–2 weeks according to DNA content. Derivation of the haNSCLCs and WT‐NSCLCs was performed according to a previous protocol.^[^ ^19^ ^]^

### 10× Genomics Single‐Cell RNA‐Seq

The haNSCs were dissociated into single cells, and washed with PBS. The centrifuged cell pellet was resuspended in DMEM (Thermo, 12800017, USA). The cell viability was determined using the LUNA‐II™ (Logos Biosystems, Korea), with density being adjusted to 500–600 cells µL^−1^. The cell suspension was loaded onto the Chromium single cell controller (10× Genomics). Gel Bead Kit v3.1 (10× Genomics, 1000075, USA) and Chromium Single Cell B Chip Kit (10× Genomics, 1000074, USA) were utilized to generate single‐cell gel beads in the emulsion according to the manufacturer's protocol. A Previous literature was referred to for further detailed methods.^[^ ^33^ ^]^ This sequencing was performed at Kidio Technology Services Company (Guangzhou, China).

### Electrophysiology of the haNSCs

The ruptured‐patch whole‐cell configuration of the patch‐clamp recordings was carried out at room temperature utilizing an EPC10 USB amplifier (HEKA Elektronik) with a sampling frequency of 10 kHz. Patch pipette electrodes were fashioned from borosilicate glass using a micropipette puller (P‐97 model, Sutter Instrument). These electrodes exhibited a resistance of approximately 3–5 MΩ. For the I‐V recordings, voltage pulses were administered in 10‐mV steps in the range of ‐80 mV to +70 mV, each lasting 500 ms and with a holding potential of −60 mV. The extracellular solution comprised 141 mm NaCl, 4.7 mm KCl, 3.0 mm MgCl_2_·6H_2_O, 1 mm EGTA, 10 mm HEPES, and 10 mm glucose, adjusted to pH 7.4. The pipette solution contained 125 mm KCl, 4 mm MgCl_2_·6H_2_O, 10 mm HEPES, 10 mm EGTA, and 5 mm Na_2_ATP and was adjusted to pH 7.4.

### Subtype Differentiation of haNSCs

Neuronal differentiation of haNSCs was induced in Ndiff medium supplemented with 20 ng mL^−1^ BDNF (PeproTech, AF‐450‐02, USA) and 20 ng mL^−1^ NT‐3 (PeproTech, AF‐450‐03, USA) for approximately three weeks. For astrocyte differentiation, haNSCs were induced in Ndiff medium supplemented with 1% FBS and 10 ng mL^−1^ BMP4 (PeproTech, AF‐120‐05ET, USA) for one week.

### Vector Construction and Transfection

The PB and PBase plasmids were constructed as described by the group previously with slight modification.^[^ ^14^ ^]^ For KO plasmids, sgRNAs with specific sequences were designed using an online tool (http://crispor.tefor.net/crispor.py). Their oligonucleotides were phosphorylated using T4PNK (Takara, 2021A, Japan), annealed, and ligated onto a linearized modified pSpCas9(BB)−2A‐GFP (PX458) (Addgene, 48138). For OE vectors, the coding DNA sequence (CDS) of the desired gene was fused to the modified PB vector (SBI, PB513B‐1). The electroporation conditions were 1400 V, 13 ms, and 3 pulses.

### Immunostaining and Karyotype Analysis

Immunostaining and karyotype analysis were performed as described previously.^[^ ^19^ ^]^ The primary antibodies used are as follows: PAX6 (ABclonal, A7334, USA), SOX1 (CST, 4194S, USA), NESTIN (ABclonal, A11861, USA), GFAP (Abcam, ab7260, UK), Tuj1 (Abcam, ab7751, UK), MAP2 (Abcam, ab32454, UK), NeuN (Abcam, ab104224, UK) and SERT (Abcam, ab130130, UK). The secondary antibodies used are as follows: 594 rabbit anti‐mouse (Yeasen, 33912ES60, China), FITC goat anti‐mouse (ABclonal, AS001, USA), Cy3 goat anti‐rabbit (ABclonal, AS007, USA), and 594 donkey anti‐goat (Yeasen, 34312ES60, China). Nuclei were stained with DAPI (Yeasen, 40727ES10, China). Images were captured utilizing a TCS SP8 confocal laser scanning microscope (Leica, Germany).

### Induction of Cerebral Organoids

The induction of cerebral organoids was performed as described previously with slight modifications.^[^ ^34^, ^35^ ^]^ Initially, haNSCs or ESCs were trypsinized into single‐cells. Then the cells were resuspended in Ndiff medium and floating cultured to aggregate within 24 h. Then the Ndiff medium was supplemented with 2 µm SB‐431542 (MCE, HY10431, China) and DMH1(MCE, HY12273, USA) for 7 days, with medium half‐changed daily. After 7 days of culture, the aggregates were transferred to Matrigel (BD, 354230, USA) pre‐coated six‐well plates for adherent culture, with medium changed every day. Adherent culture was continued until the appearance of rosette‐like structures. Subsequently, the neural tube‐like structures were dissected with needles. The cells derived from the neural tube‐like structures were floating cultured in a medium composed of Ndiff medium supplemented with 2 µm SB‐431542, DMH1, and 2% B27 (Gibco, 17504044, USA) in 2–3 days, allowing the self‐organization into spherical forebrain dorsal organoids.

### Establishment of Mutant Library

The PB‐trapping system (PB: PBase = 3:1) was induced to haNSCs to bring mutations. The mutant cells were Selected with 1 µg mL^−1^ puromycin (MCE, HY‐B1734A, USA), which were expanded and ready for H_2_O_2_ treatment. Both non‐mutant and mutant groups were treated with H_2_O_2_. The survived cells from mutant group were expanded and prepared as an oxidative stress‐resistant mutation library (ML).

### CCK‐8, GSH, and SOD Assays and Apoptosis Detection

Cell viability was determined using the Cell Counting Kit‐8 (Yeasen, 40203ES76, China). A total of 5 × 10^3^ cells from each sample were seeded in a well of a 96‐well plate, cultured overnight, and incubated with CCK8 for 4 h followed by measurement with an enzyme‐labeled instrument. GSH levels and SOD activities were measured using a GSH Assay Kit (Beyotime, S0053, China) and a Total Superoxide Dismutase Assay Kit (Beyotime, S0101M, China), respectively, according to the manufacturers’ protocols.

To assess oxidative stress resistance, cells were seeded in one well of a 6‐well plate at a density of 2×10.^5^ The cells were then treated with 0.8 mm H_2_O_2_ for 4 h. Thereafter, trypsinized single cells were processed with an Annexin V‐Alexa Fluor 488/PI Apoptosis Detection Kit (Yeasen, 40305ES60, China) or DRAQ7 (MK Bio, MX4237, China) according to the manufacturers’ instructions. Well‐prepared samples were analyzed by flow cytometry. FACS data were analyzed using FlowJo software (San Carlos, USA).

### Quantitative Real‐Time PCR

RNA was extracted with TRIzol reagent (Thermo, 317110, USA) and reverse transcribed to cDNA using a Prime Script TM RT Reagent Kit (Takara, RR047A, Japan). Quantitative PCR was conducted on a BioRad CFX touch 96 instruments with Hieff qPCR SYBR Green Master Mix (No Rox) (Yeasen, 11201ES08, China). The *Gapdh* gene was used for normalization of the expression level. The presented data represent the mean ± SD of three independent experiments.

### Analysis of the Splinkerette PCR Data, RNA‐Seq Data, and CGH Data

The Splinkerette PCR conditions and the respective analysis methods were described in detail in a previous report.^[^ ^14^ ^]^ All RNA and DNA samples were sent to a local company for sequencing. The RNAseq and CGH data analysis methods were performed according to a previous report.^[^ ^18^ ^]^

### Ethics Approval

All animal‐related experiments were performed according to the guidelines of the Animal Care and Use Committee of Nankai University.

## Conflict of Interest

The authors declare no conflict of interests.

## Supporting information

Supporting Information

## Data Availability

The data that support the findings of this study are available from the corresponding author upon reasonable request. The RNA‐seq, 10× Genomics, and screening raw dataset in this study have been deposited in the Genome Sequence Archive of the Beijing Institute of Genomics (BIG) Data Center with accession numbers CRA015861.
