# Supplementary material for: Genetic Screening of Haploid Neural Stem Cells Reveals that Nfkbia and Atp2b4 are Key Regulators of Oxidative Stress in Neural Precursors
Source: Adv Sci (Weinh). 2024 Apr 26;11(29):2309292. doi: 10.1002/advs.202309292 (PMC11304298; doi:10.1002/advs.202309292)
Supplement: Supplementary file 1 — Supporting Information [file ADVS-11-2309292-s001.pdf]

ADVANCED SCIENCE
Open Access

Supporting Information

for *Adv. Sci.*, DOI 10.1002/advs.202309292

Genetic Screening of Haploid Neural Stem Cells Reveals that *Nfkbia* and *Atp2b4* are Key Regulators of Oxidative Stress in Neural Precursors

Shaochen Nie, Wenhao Zhang, Xin Jin, Xiaoyan Li, Shengyi Sun, Yiding Zhao, Qingshen Jia, Luyuan Li, Yan Liu, Dayong Liu* and Qian Gao**

Supporting Information

Title: Genetic Screening of Haploid Neural Stem Cells Reveals that *Nfkbia* and *Atp2b4* are Key Regulators of Oxidative Stress in Neural Lineages

Running title: A Haploid Cell Line for Neural Functional Genetics

Authors and affiliations:

Shaochen Nie, Wenhao Zhang, Xin Jin, Xiaoyan Li, Shengyi Sun, Yiding Zhao, Qingshen Jia, Luyuan Li, Yan Liu, Dayong Liu* and Qian Gao**

S. Nie, X. Li, D. Liu*

Department of Endodontics & Laboratory for Stem Cells and Endocrine Immunology

Tianjin Medical University School of Stomatology

Tianjin 300070, China.

E-mail: dyliuperio@tmu.edu.cn (D.L.)

W. Zhang, X. Jin, S. Sun, Y. Zhao, Q. Jia, L. Li, Q. Gao*

State Key Laboratory of Medicinal Chemical Biology and College of Pharmacy

Nankai University

Tianjin 300350, China.

E-mail: gaoqian@nankai.edu.cn (Q.G.)

Q. Gao*

Tianjin Key Laboratory of Animal and Plant Resistance

College of Life Sciences

Tianjin Normal University, Tianjin, China.

E-mail: gaoqian@nankai.edu.cn (Q.G.)

X. Jin

School of Medicine

Nankai University

Tianjin 300071, China.

Y. Liu

Department of Obstetrics

Tianjin First Central Hospital

Nankai University, Tianjin 300192, China.

E-mail: 30819007@nankai.edu.cn (Y.L.);

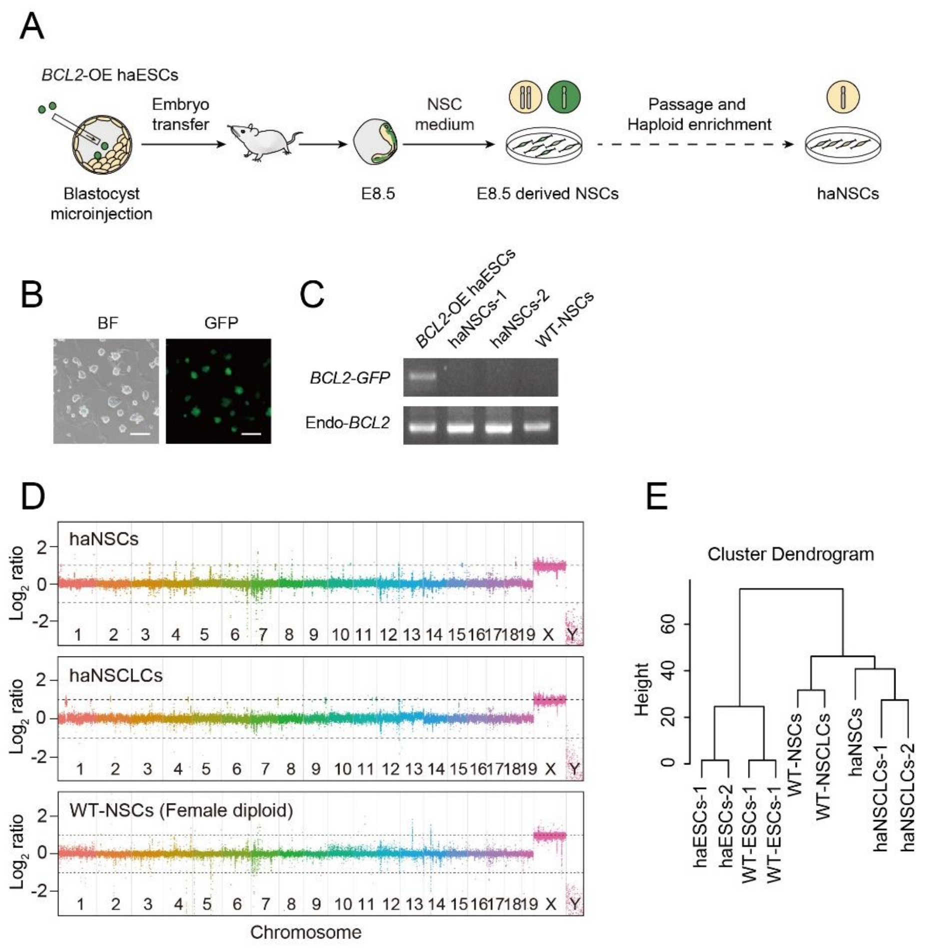

Figure S1. The establishment of haNSCs (related to Figure 1).

A) A schematic overview of the establishment of haNSCs from mouse E8.5 embryos.

B) Morphology of GFP-labeled *BCL2*-OE haESCs in the bright field and FITC channels. Scale bar, 100 μm.

C) The genotyping results of *BCL2*-OE haESCs, haNSCs and WT-NSCs. The exogenous *BCL2* was not detected in haNSCs.

D) CNV analysis of haNSCs, haNSCLCs and WT-NSCs (Female diploid) with male 129Sv/Jae kidney DNA as a reference control.

E) Transcriptome cluster analysis of haESCs, WT-ESCs, WT-NSCs, WT-NSCLCs, haNSCs and haNSCLCs.

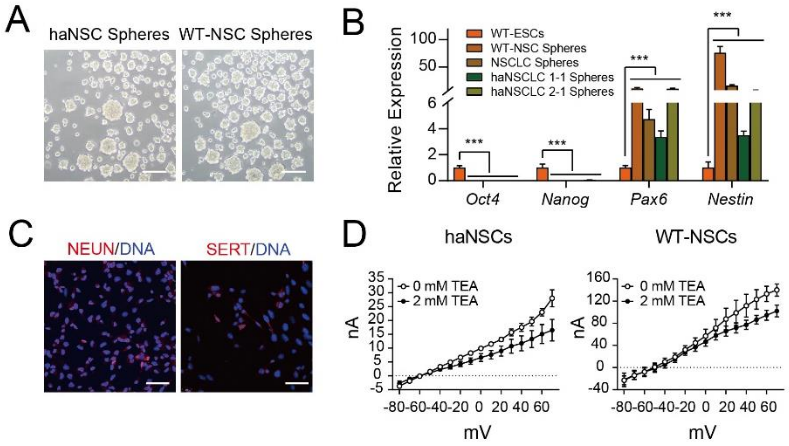

Figure S2. Characterization of haNSCs (related to Figure 2).

A) Morphologies of the neural spheres derived from haNSCs and WT-NSCs. Scale bar, 100 μm.

B) Expression levels of *Oct4*, *Nanog*, *Pax6* and *Nestin* in WT-ESCs, WT-NSC spheres, NSCLC spheres and haNSC spheres. t test, ***$p < 0.001$. Data were presented as the mean ± SEM.

C) Immunostaining of neuronal-specific markers (NEUN and SERT) in neurons derived from haNSCs. Scale bar, 50 μm.

D) The current-voltage relationship in the absence and presence of TEA. Data represented three independent experiments.

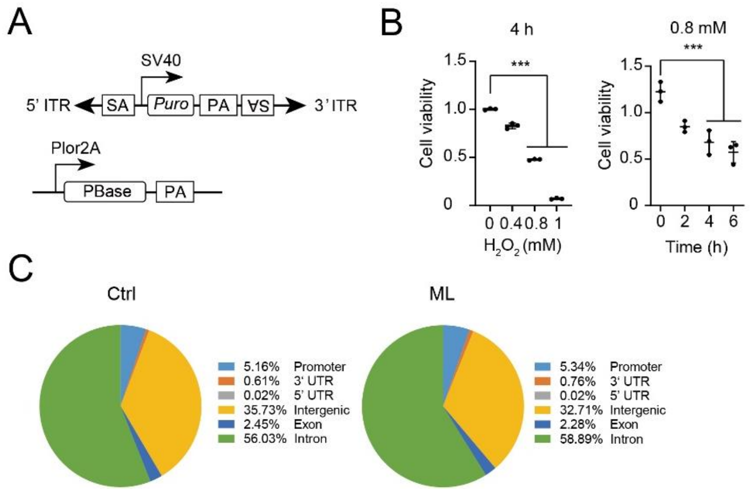

Figure S3. Genetic screening with haNSCs (related to Figure 3).

A) Schematic diagram of PB trapping vectors. The PB vector contained 5' ITR and 3' ITR, the inverted terminal repeats of PB. SA, splice acceptor; SV40, promoter; *Puro*, the coding sequence of the *Puro* resistance gene; PA, poly (A) sequence. The PBase vector contained Plor2A (promoter), a PBase coding sequence and PA.

B) Viability of haNSCs treated with 0, 0.4, 0.8 and 1 mM H_2O_2 for 4 h and with 0.8 mM H_2O_2 for 0, 2, 4 and 6 h. Data represented three independent experiments. t test, ***$p < 0.001$. Data were presented as the mean ± SD.

C) Analysis of the integration sites after H_2O_2 selection over genomic regions: promoters (1 kb upstream of the transcription starting sites), intragenic regions and intergenic regions in Ctrl and ML cells.

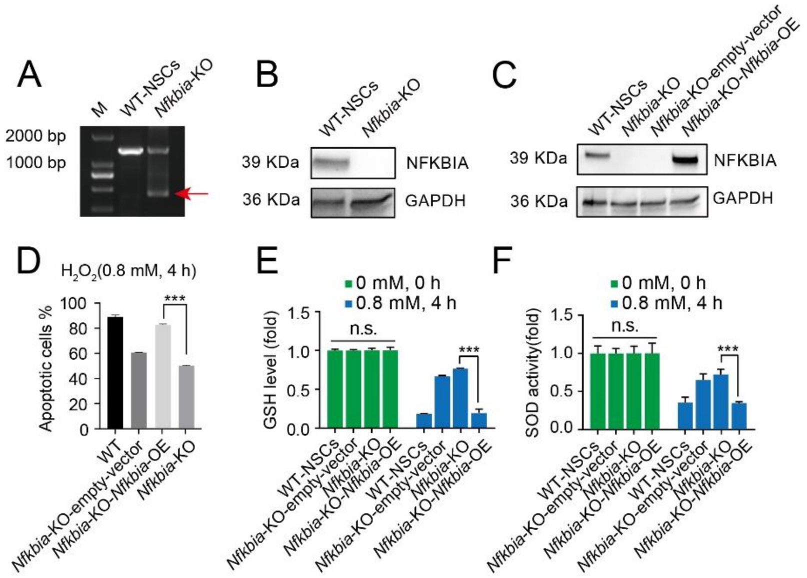

Figure S4. Validation of *Nfkbia*-KO in WT-NSCs (related to Figure 4).

A) Genotype identification of *Nfkbia*-KO NSCs.

B) Western blotting to detect NFKBIA in WT-NSCs and *Nfkbia*-KO NSCs. GAPDH was used as a loading control.

C) Western blotting to detect NFKBIA in WT-NSCs, *Nfkbia*-KO NSCs, *Nfkbia*-KO-empty-vector NSCs and *Nfkbia*-KO-*Nfkbia*-OE NSCs. GAPDH was used as a loading control.

D) Apoptosis analysis of WT-NSCs, *Nfkbia*-KO NSCs, *Nfkbia*-KO-empty vector NSCs and *Nfkbia*-KO-*Nfkbia*-OE NSCs after treatment with 0.8 mM H_2O_2 for 4 h by DRAQ7 analysis. Data represented three independent experiments. t test, ****p* < 0.001, n.s. not significant. Data were presented as the mean ± SD.

E) GSH levels in WT-NSCs, Nfkbia-KO NSCs, *Nfkbia*-KO-empty vector NSCs and *Nfkbia*-KO-*Nfkbia*-OE NSCs after treatment with/without 0.8 mM H_2O_2 for 4 h. The data represented three replicates. t test, ****p* < 0.001, n.s. not significant. Data were presented as the mean ± SD.

F) SOD activities in WT-NSCs, Nfkbia-KO NSCs, *Nfkbia*-KO-empty-vector NSCs and *Nfkbia*-KO-*Nfkbia*-OE NSCs after treatment

with/without 0.8 mM H_2O_2 for 4 h. The data represented three replicates. t test, ***$p < 0.001$, n.s. not significant. Data were presented as the mean ± SD.

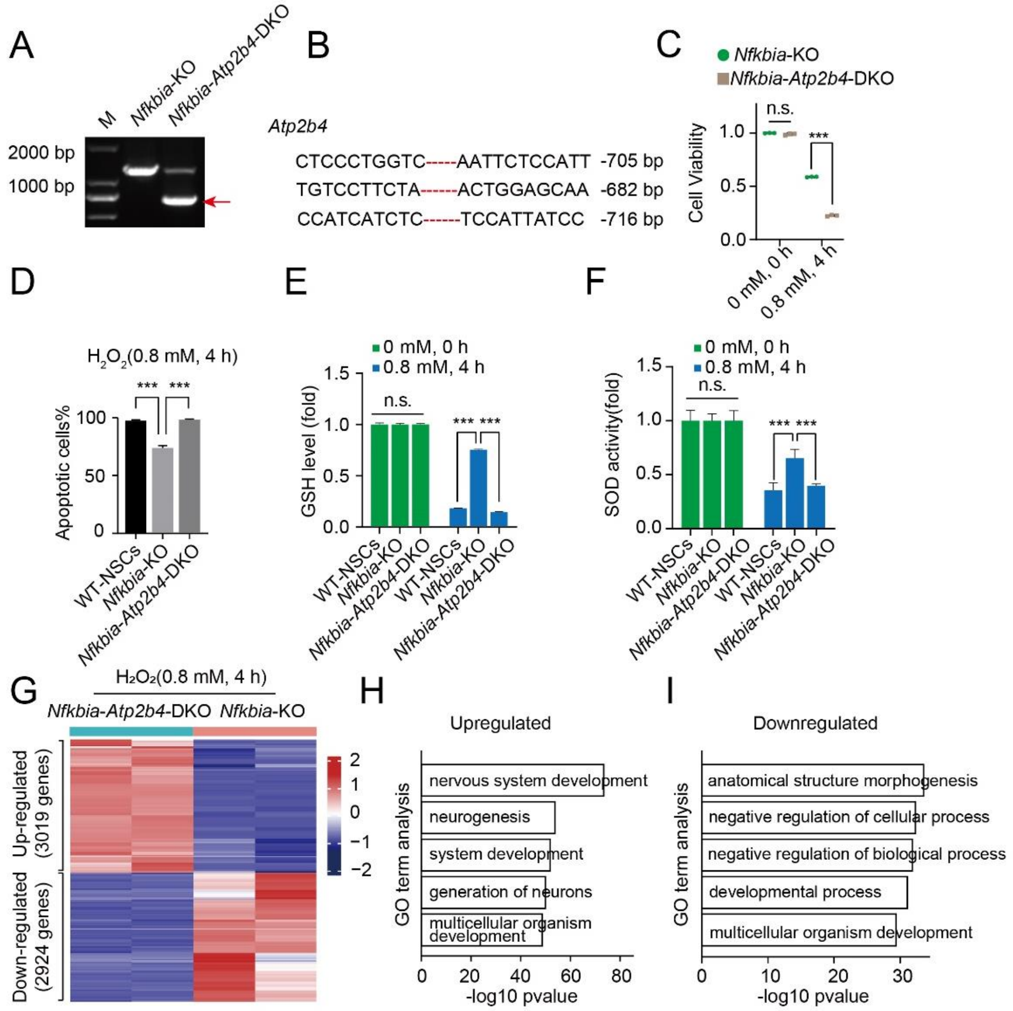

Figure S5. Analysis of *Nfkbia*-related genes involved in oxidative stress (related to Figure 5).

A) Genotype identification of *Nfkbia-Atp2b4*-DKO NSCs.

B) Genotype sequencing results for *Nfkbia-Atp2b4*-DKO NSCs.

C) CCK-8 assay results with *Nfkbia*-KO NSCs and *Nfkbia-Atp2b4*-DKO NSCs after treatment with 0.8 mM H_2O_2 for 4 h. Data represented three independent experiments. t test, ***p < 0.001. Data were presented as the mean ± SD.

D) Apoptosis analysis of WT-NSCs, *Nfkbia*-KO NSCs and *Nfkbia-Atp2b4*-DKO NSCs after treatment with 0.8 mM H_2O_2 for 4 h by PI/Annexin V assay. Data represented three independent experiments. t test, ***p < 0.001, n.s. not significant. Data were presented as the mean ± SD.

E) GSH levels in WT-NSCs, *Nfkbia*-KO NSCs and *Nfkbia-Atp2b4*-DKO NSCs after treatment with/without 0.8 mM H_2O_2 for 4 h. Data represented three independent experiments. t test, ***$p < 0.001$, n.s. not significant. Data were presented as the mean ± SD.

F) SOD activities in WT-NSCs, *Nfkbia*-KO NSCs and *Nfkbia-Atp2b4*-DKO NSCs after treatment with/without 0.8 mM H_2O_2 for 4 h. Data represented three independent experiments. t test, ***$p < 0.001$, n.s. not significant. Data were presented as the mean ± SD.

G) Heatmap of DEGs among *Nfkbia*-KO NSCs and *Nfkbia-Atp2b4*-DKO NSCs after treatment with/without 0.8 mM H_2O_2 for 4 h.

H) GO analysis of the upregulated genes in *Nfkbia-Atp2b4*-DKO NSCs compared with *Nfkbia*-KO NSCs after treatment with/without 0.8 mM H_2O_2 for 4 h.

I) GO analysis of the downregulated genes in *Nfkbia-Atp2b4*-DKO NSCs compared with *Nfkbia*-KO NSCs after treatment with/without 0.8 mM H_2O_2 for 4 h.

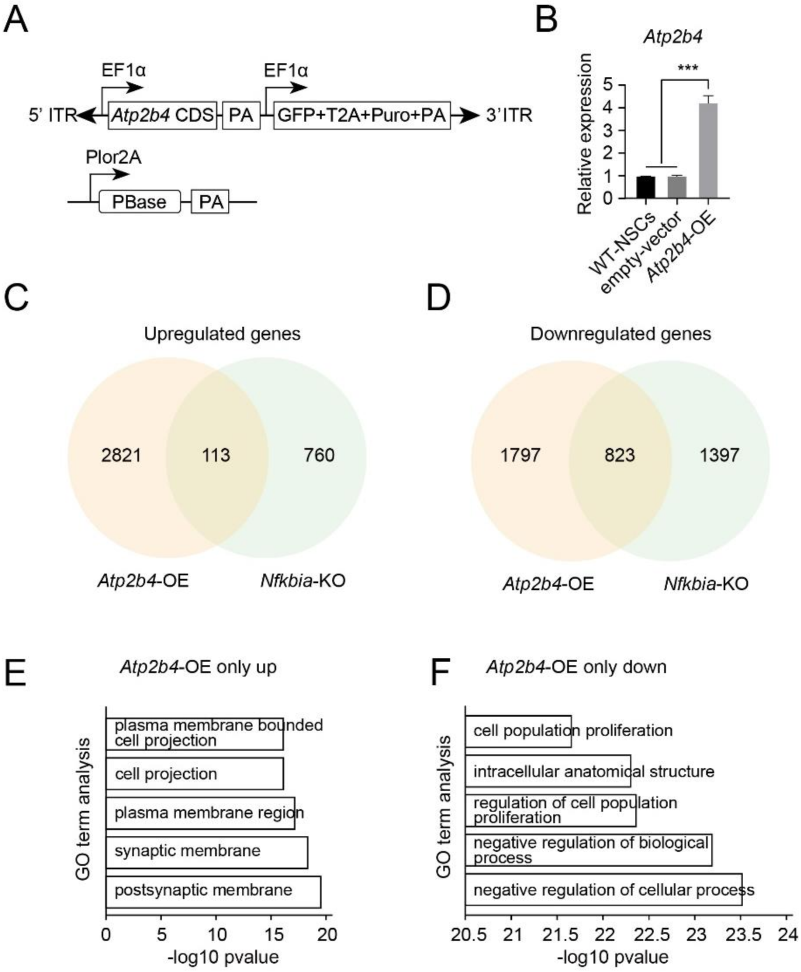

Figure S6. Validation of the involvement of *Atp2b4* in oxidative toxicity resistance (related to Figure 6).

A) Schematic overview of the vectors used for *Atp2b4* OE.

B) The expression levels of *Atp2b4* in WT-NSCs, empty-vector NSCs and *Atp2b4*-OE NSCs according to qPCR. Data represented three independent experiments. t test, ***$p < 0.001$, n.s. not significant. Data were presented as the mean ± SD.

C) Venn diagram depicting the upregulated DEGs between *Nfkbia*-KO NSCs and *Atp2b4*-OE NSCs. The yellow circle represented the overlapping upregulated DEGs between *Nfkbia*-KO NSCs and *Atp2b4*-OE NSCs. The pink circle represented the DEGs upregulated in *Atp2b4*-OE NSCs only. The green circle represented the DEGs upregulated in *Nfkbia*-KO NSCs only.

D) Venn diagram illustrating the downregulated DEGs between *Nfkbia*-KO NSCs and *Atp2b4*-OE NSCs. The yellow circle represents the overlapping downregulated DEGs between *Nfkbia*-KO NSCs and *Atp2b4*-OE NSCs. The pink circle represents the DEGs downregulated in *Atp2b4*-OE NSCs only. The green circle represents the DEGs downregulated in *Nfkbia*-KO NSCs only.

E) GO analysis is performed on the subset of DEGs that are upregulated in *Atp2b4*-OE NSCs only.

F) GO analysis is according to the subset of DEGs that are downregulated in *Atp2b4*-OE NSCs only.

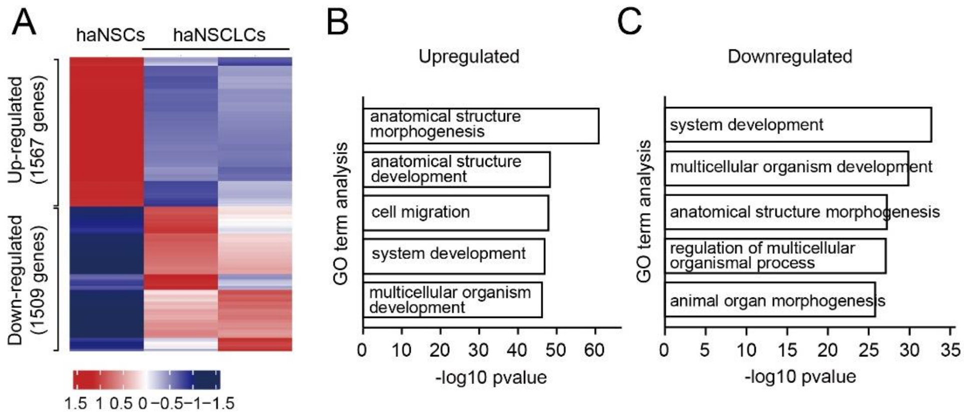

Figure S7. Comparation between haNSCs and haNSCLCs at the transcriptome level (related to Figure 1).

A) Heatmap of DEGs between haNSCs and haNSCLCs.

B) GO analysis of the upregulated genes in haNSCs when compared with haNSCLCs.

C) GO analysis of the downregulated genes in haNSCs when compared with haNSCLCs.

Table S1. Primer Sequences (related to Figure 3, 4, 5, 6, S1, S2, S4 and S5).

Trapping vector	*Puro*-PA F	GAATCCGGAATGACCGAGTACAAGCCC
	Puro-PA R	GTTACCGGTGCAGTGAAAAAAATGCTTTATT TGTG
	EF1α F	CTAGCAAGGATCTGCGATCGCTCCG
	EF1α R	TCCGGAGTAGGCGCCGGTCACAGCTT
SPLNK PCR	SPLNK-GATC-TOP	GATCCCACTAGTGTCGACACCAGTCTCTAAT TTT TTTTTTCAAAAAAA
	SPLNK-BOT	CGAAGAGTAACCGTTGCTAGGAGAGACCGT GGC TGAATGAGACTGGTGTCGACACTAGTGG
	SPLNK#1	CGAAGAGTAACCGTTGCTAGGAGAGACC
	SPLNK#2	GTGGCTGAATGAGACTGGTGTCGAC
	3'SPLNK-PB#1	GTTTGTTGAATTTATTATTAGTATGTAAG
	3'SPLNK-PB#2	CGATAAAACACATGCGTC
	3'SPLNK-PB-SEQ	ACGCATGATTATCTTTAAC
	5'SPLNK-PB#1	ACCGCATTGACAAGCACG
	5'SPLNK-PB#2	CTCCAAGCGGCGACTGAG
	5'SPLNK-PB-SEQ	CGACTGAGATGTCCTAAATGC
Nfkbia-KO	*Nfkbia* sg1-1	CACCGGGTCAAAGAGGGCACCCGCG
	Nfkbia sg1-2	AAACCGCGGGTGCCCTCTTTGACC C
	Nfkbia sg2-1	CACCGCGTTAGTTTACGGCTGCATC
	Nfkbia sg2-2	AAACGATGCAGCCGTAAACTAACG C
	Nfkbia sg3-1	CACCG AGCCTCTATCCACGGCTACC
	Nfkbia sg3-2	AAAC GGTAGCCGTGGATAGAGGCTC
	Nfkbia sg4-1	CACCG GGCAGACCTACCATTGTAGT
	Nfkbia sg4-2	AAAC ACTACAATGGTAGGTCTGCCC
Atp2b4-KO	*Atp2b4* sg1-1	CACCGGCTGCAGAGTCGCATCGAAC
	Atp2b4 sg1-2	AAACGTTCGATGCGACTCTGCAGCC
	Atp2b4 sg2-1	CACCGTCCACCCGGAGGTCGGTAGA
	Atp2b4 sg2-2	AAACTCTACCGACCTCCGGGTGGAC
Genotype	*Nfkbia* genotype F	CAATCATCCACGAAGAGAAGCC
	Nfkbia genotype R	TAGAACAGCCGTCAGCGTG
	Atp2b4 genotype F	CTGCATCGTGGTGGTAGCA
	Atp2b4 genotype R	AGGCTTTCAGAAACTCCCAGC
qPCR	*Gapdh* F	AGGTCGGTGTGAACGGATTTG
	Gapdh R	TGTAGACCATGTAGTTGAGGTCA
	Oct4 F	TGCAGCTCAGCCTTAAGAACATG
	Oct4 R	CCACCTCACACGGTTCTCAATG
	Nanog F	CAGCCTCCAGCAGATGCAAG
	Nanog R	TGCTGGGATACTCCACTGGTG

	Pax6 F	AGCTTCACCATGGCAAACAACC
	Pax6 R	CTGACTGTTCATGTGTGTTTGCATGTG
	Nestin F	TCGCTTGCAGACACCTGGAAG
	Nestin R	GTCACAGGAGTCTCAAGGGTATTAGG
Nfkbia-OE	*Nfkbia* CDS-F	TAGAGCTAGCGAATTATGTTTCAGCCAGCTGGG
	Nfkbia CDS-R	GCGCGGCCGCGGATCTTATAATGTCAGACGCTGGCC
Atp2b4-OE	*Atp2b4* CDS-F	TAGAGCTAGCGAATTATGACGAATCCACCAGGACAAAGC
	Atp2b4 CDS-R	GCGCGGCCGCGGATCTCAGACCGGTGTCTCCAGGC
